# Characteristics and management of municipal solid waste in Uyo, Akwa Ibom state, Nigeria

**DOI:** 10.1038/s41598-024-61108-0

**Published:** 2024-05-14

**Authors:** Uduak Bassey, Abasi-ofon Tom, Udemeobong Okono, Mbetobong John, Maja Sinn, Ayoge Bassey, Uduak Luke, Satyanarayana Narra

**Affiliations:** 1 Department of Waste and Resource Management, Faculty of Agricultural and Environmental Sciences, University of Rostock, 18051 Rostock, Germany; 2 Berlin School of Technology, SRH Berlin University of Applied Science, 10587 Berlin, Germany; 3 University of Uyo, Uyo, Akwa Ibom State Nigeria; 4 School of Chemistry, University of Glasgow, G128QQ Scotland, United Kingdom; 5 Max Planck Institute of Molecular Physiology, Department of Systemic Cell Biology, 44227 Dortmund, Germany; 6 Orchid Springs Limited, Uyo, Nigeria

**Keywords:** Waste-composition, Waste-management, Municipal-solid-waste, Waste-forecast, Recycling, Waste-collection, Environmental sciences, Sustainability, Environmental impact

## Abstract

Increased urbanization and population lead to increased consumption of manufactured goods. This ultimately results in increased production of waste. Identifying its composition is crucial for planning an effective solid waste management strategy. This study assesses the characteristics and composition of the waste generated within the Uyo Capital City Development Area of Akwa Ibom State, Nigeria. This is to aid in developing a scientifically supported waste management pilot system for the state. Direct waste sorting and characterization were conducted on the municipal solid waste arriving at the landfill during the study period. Over 50% of the generated wastes are recyclables and composed of plastics, metals, and paper, while the fraction of organic waste is over 30%. Similarly, the waste generation per capita is 1.34 kg/person/day, while the generation forecast over the next ten years is estimated to increase by approximately 40%. Furthermore, over 9,000 surveys were completed by residents to establish a problem statement about the existing waste collection and disposal system, and possible solutions. Importantly, a majority of survey respondents were willing to source-separate their wastes and supported paying a fee for adequate waste collection. This strongly indicates that an integrated waste management system could be established to generate value from the collected waste. Supplementary revenue can be generated through composting, recycling, and land reclamation.

## Introduction

**Figure 1:**
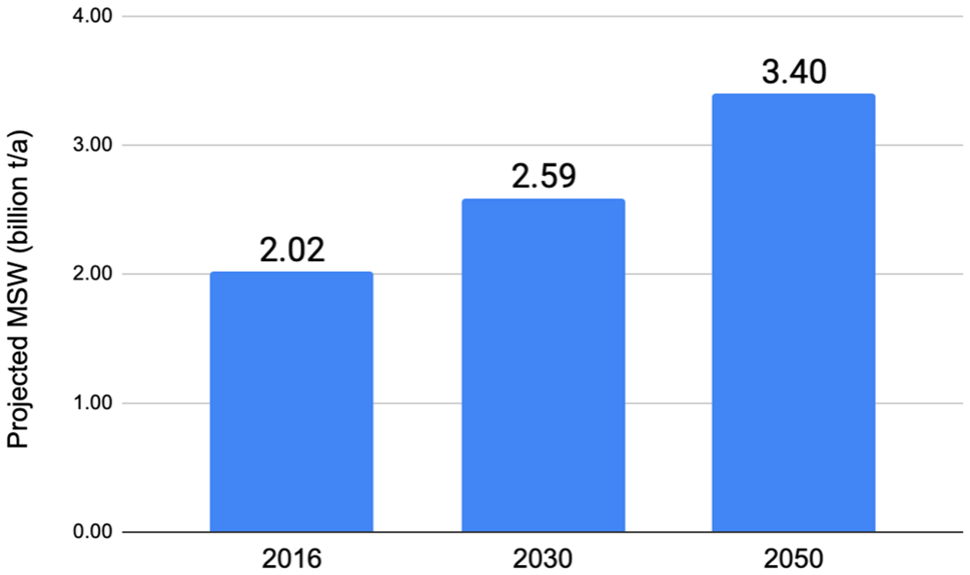
Projected generation of municipal solid waste worldwide from 2016 to 2050 (in billion metric tons) (source: Statista 2023).

Increasing population and rural-to-urban migration in developing countries is bound to result in increased municipal solid waste (MSW) generation, an effect already established in Nigeria. The annual worldwide MSW generation is projected to increase steadily from about 2.0 billion metric tons in 2016 to 3.4 billion metric tons in 2050 as shown in Fig. 1. Similarly, Nigeria generates about 25 million tons of municipal solid waste annually, and this number is expected to double by 2040^1^. Several waste management methods are practiced around the world today. Waste, by definition unwanted or unusable materials, can range from solids to liquids and gases. Municipal solid waste consists of unwanted solid remains retrieved from household & office residents, and retail and commercial business establishments in a municipality. MSW poses a great challenge with regards to its management and has been identified as one of the major challenges to reaching sustainability targets^3^. Several classes of municipal solid wastes, based on the sources of the waste generation, have been presented in literature^4–8^. Across regions and municipalities, there is great variation of MSW in composition and it can be generally divided into biodegradable and non-biodegradable components. Nevertheless, typical MSW streams consist of metals, rubbers and plastics, kitchen waste, glass waste, yard waste, electronic waste, paper, cardboard, and others^2^. In Nigeria, very limited literature on the characteristics of MSW exists, as any existing effort is hampered by the difficulty in management of waste. These sources have been attributed to improper waste disposal, inefficient method of waste collection and insufficient coverage of waste existing collection systems^9^. Furthermore, the rate of waste generation in Nigeria has been relatively unknown as a result of limited studies; however, a decade and a half ago, it was reported that the rate of waste generation is Nigeria was in the range between 0.44 and 0.66 kg/capita/day with the waste density ranging between 200 and 400 kg/m^3,9,10^. Ever since, there has been some reluctance in characterizing the wastes generated in Nigeria. However, with the population of Nigeria increasing at an incline, coupled with increased industrialization and commercialization of Nigeria’s economy, it has been noted that more waste is also being generated^11,12^. Consequently, this study seeks to address the waste management situation in Nigeria by analyzing characteristics and composition of the waste generated in the city of Uyo in Akwa Ibom state. The specific waste management methods are reviewed in the next section, and the MSW of the study area is characterized following the methodology described below. Furthermore, suggestions on ways of improving waste management in Uyo are presented.

### Municipal solid waste management strategies

MSW management approaches in different regions and countries are connected to the Gross Domestic Product (GDP), income and population of the assessed country. It describes the process of waste management from generation to disposal. Hence, there is significant variation in MSW management between developed and developing countries. Nanda & Berutti^2^ summarizes the MSW management stages as:

i. Waste generation;
ii. Waste retrieval (collection) and handling; and
iii. Waste disposal, including waste treatment and processing.

Furthermore, MSW management methods are summarized as:

i. Mechanical recycling or diversion;
ii. Waste-to-energy (WTE) conversion;
iii. Landfilling;
iv. Incineration; and
v. Composting

The following section summarizes the principles of the methods highlighted above.

#### Mechanical recycling

Mechanical recycling involves the conversion of solid waste into a purified or different form without necessarily altering the chemical composition of the parent material^13^. A typical instance could be the integration of ground water sachets (made from low-density polyethylene materials) into molded bricks to improve brick strength and toughness. These granules from the water packaging material represent a form of recycled material devoid of alteration in chemical composition. The application of similar sorts of recycling methods is limited, hence, this method of MSW measurement is uncommon and not suitable to make use of a significant amount of the MSW bulk.

#### Waste-to-energy conversion

This is the transformation of waste to useful energy such as electrical, biological, chemical and others. There exist various methods of converting waste to energy such as organic or food waste using biogas, plastics and other combustibles using thermochemical methods and to solid residual fuels (SRFs). Popular thermochemical conversion methods include pyrolysis and gasification. Pyrolysis involves thermal degradation of materials at high temperatures in the absence of oxygen. Typical pyrolysis products depend on the feed stream composition and usually include pyrolysis oil, char, tar, and gases^6^. Detailed descriptions of pyrolysis of MSW are presented in literature^14–20^. On the other hand, gasification is a process whereby a carbon-containing material (CCM) is converted into syngas under limited oxygen conditions and at high temperatures. Like pyrolysis, product composition depends on the composition of the feed stream^6^. Detailed description of thermochemical treatment is presented in literature^21–33^. Various variations in the thermochemical processes mentioned exist, and a detailed description of these variations are presented in literature^6^. It is worthy to note that these technologies are common in developed economies and are gradually being introduced in developing ones.

#### Incineration

Incineration is a widely used method to treat waste due to its potential of reducing waste by over 90% volume^6^. It is the combustion of waste materials in the presence of oxygen and is usually performed in specially designed incineration plants in developed countries. In underdeveloped countries, this can be performed in open dumps. While it usually presents a cheaper mode of waste destruction, it is strongly plagued by environmental pollution, i.e., the release of harmful substances and toxins, and is hence not an advisable method except when sufficient pollution abatement procedures are put in place^34,35^. MSW incineration has been reported as an energy recovery method, although this is no longer commonly practiced. However, sufficient literature on MSW incineration exists^36–38^.

#### Landfilling

**Figure 2:**
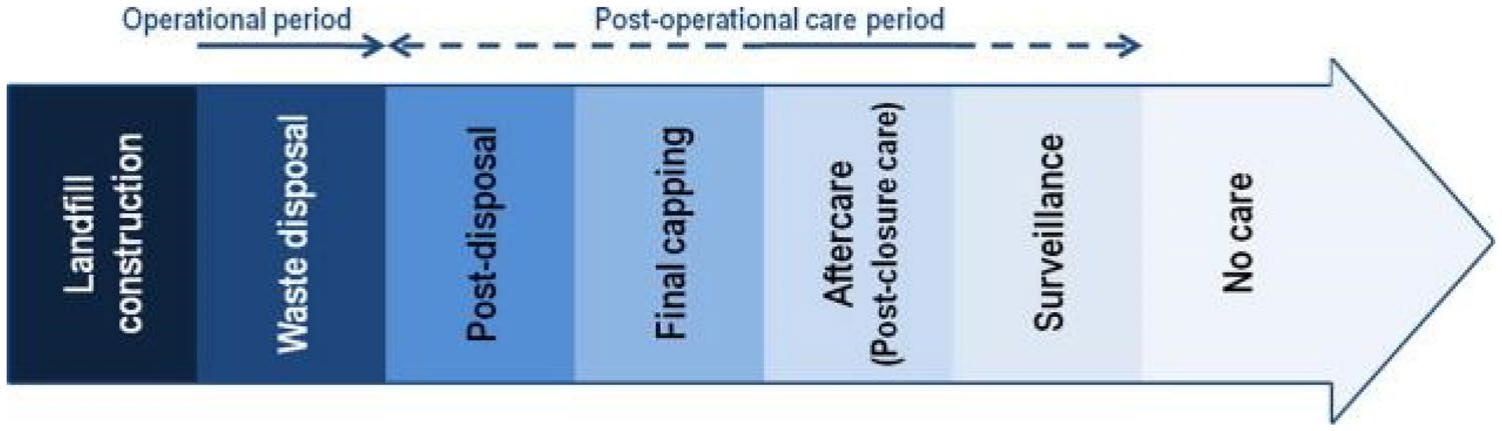
Various management phases over MSW landfill life cycle. Adapted from Laner et al.^44^.

Landfilling has been a dominant MSW disposal method, which stems from the comparatively high cost of alternative treatment or disposal alternatives. Similarly, this has been the dominant waste disposal method in developing countries^39^. It refers to the process of dumping solid waste on a site reserved for such purposes. There exist various classifications of landfills depending on the source of waste, e.g. a MSW landfill. Some additional features that may be integrated into the management process include equipment, staff, high-level control engineering, pollution abatement controls, leachate containment capabilities, etc. Various classifications exist, and these are based on conventions defined using characteristics of the landfill. The prominent classifications are those set out by the Malaysian Ministry of Housing and Local Government and the United Nations. More details for this convention can be obtained from literature^40–42^. A summary of the modified application of the classification system adapted from Idowu et al., 2019 is presented in Table 1 below. While landfills have existed from early ages, the concept has been modernized in well-managed and engineered facilities for solid waste disposal^43^. Another aspect of modern landfills is monitoring and management of landfills after closure with particular focus on aftercare strategies. A full description of this can be seen in literature^44^. A summary of the management procedures over the lifetime of a landfill is illustrated in Fig. 2.

**Table 1:** Modified application of the classification system (adapted from Idowu et al., 2019^42^).

Level of control	Level	Management and operation	Operational facility
None	0	Uncontrolled dumping—no controls	Uncontrolled burning; lacking most ‘‘control” functions
Low (semi-controlled facility)	1	Site staffed; waste placed in designated area; some site equipment	Site staffed, some containment and management of combustion process; basic operating procedures to control nuisance
Medium (controlled facility)	2	Waste compacted using site equipment; waste covered (at least irregularly)	Emission controls to capture particulates; trained staff follow set operating procedures; equipment properly maintained; ash properly managed
Medium / high (engineered facility)	3	Engineered landfill site: use of daily cover material; some level of leachate containment and treatment; collection of landfill gas	High levels of engineering and process control over residence time, turbulence and temperature; emission controls to capture acid gases and capture dioxins; active management of fly ash
High (state-of the-art facility)	4	Fully functional sanitary landfill site: properly sited and designed; leachate containment (naturally consolidated clay on the site or constructed liner); leachate and gas collection; gas flaring and/or utilization; final cover; post-closure plan	Built to and operating in compliance with international best practice including e.g. EU or other similarly stringent stack and greenhouse gas emission criteria; fly ash managed as a hazardous waste using best appropriate technology

### Current state of waste management in Uyo, Akwa Ibom State

The Environmental Protection and Management Agency of the state of Akwa Ibom (AKSEPWMA) uses landfills as its main waste management method, with minimal resource recovery effort. There is no recorded home-waste collection system in place in most neighbourhoods; rather, general disposal bins are provided in central locations spread out within the capital city of Uyo. The waste containers are subsequently emptied into the landfill facility. Based on the various landfill classification types presented in Table 1, the classification of the landfill under study is Low class—Semi controlled—facility evidenced by presence of staff onsite, but absence of proper high-level control systems and devices. This section highlights the procedure for this waste management method.

### Factors affecting waste generation

Waste generation is affected by several factors. Afroz et al., in their work noted the following (with the first two as most important), using Dhaka city, Bangladesh as case study^45^:

i. Income: Here, a positive relationship was observed, and it was argued that from reason, increased income will result in greater demand for goods and services for convenience purposes.
ii. Household size: Here, a positive relationship was observed with reasonable implication that a direct proportionality exists between household size and waste generation.
iii. Willingness to separate the waste: Here, the contribution of this factor was significant and Afroz et el., presented that this could be explained by the fact that households willing to separate wastes (for reuse) at home will ultimately generate less waste.
iv. Environmental concern: Here, this factor was observed to be significant and supported the hypothesis by Afroz et al., that the respondents who cared about environmental sustainability will generate less waste and ultimately improve the waste management program.

Additionally personal attitudes and other factors like education, average living cost, cultural patterns, age structure of households, and population have also been observed in literature to affect waste generation^46–51^.

## Methodology

### Study area

**Figure 3:**
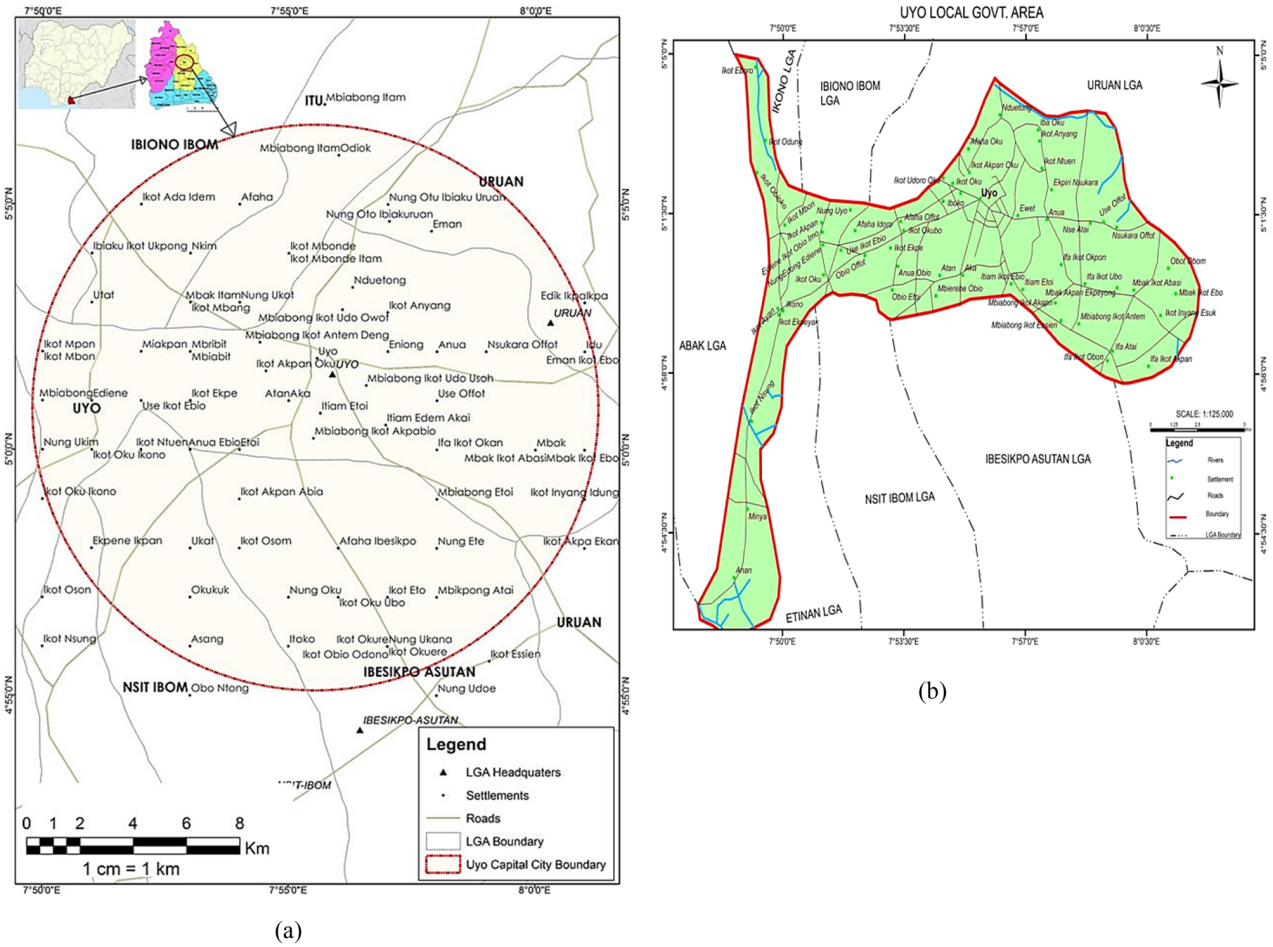
(**a**) Area within the enclosed circle indicates the mapped area of UCCDA including the contribution of the surrounding LGAs; (**b**) Map highlighting Uyo local government area^52^.

Uyo is the capital of Akwa Ibom state in the Niger Delta region of Nigeria. It lies approximately on latitudes 4°58'N and 5°04'N and longitudes 7°51'E and 8°01'E. The capital city shares a boundary to the north with Ikono, Itu and Ibiono Ibom Local Government Areas (LGA). To the east and west, it shares boundaries with Uruan and Abak LGA respectively. In the south, it is bounded by Ibesikpo-Asutan, Etinan and Nsit-Ibom LGAs (see Fig. 3b). Uyo Capital City Development Area (UCCDA) (see Fig. 3a) is made up of Uyo and parts of eight other LGAs^52^. For a detailed overview of the LGAs included in the definition of UCCDA for the purposes of this study, in which these parts of other LGAs contribute to the waste at the landfill in question, see Supplementary Table 1. The last population census in Nigeria took place in 2006. The current projected population of UCCDA is estimated to be about 1,412,000, with an average annual growth rate of 3.4%^52,53^. Uyo has a tropical humid climate with annual rainfall estimated to be 1000 mm. Additionally, there is little variation in season and temperature^53^.

### Waste characterization study design

#### Sampling design

The study was carried out at the central landfill situated along the Uyo Village Road in Uyo, Akwa Ibom State, Nigeria. This landfill, which is operated by the AKSEPWMA, serves as the destination for the disposal of all the waste generated within the Uyo metropolitan area. Therefore, it is a suitable and representative location for assessing the characteristics and management of municipal solid waste in the area.

The AKSEPWMA is the regulatory body responsible for the management of waste collection, transportation, and disposal in the city. The landfill receives waste from a variety of sources, including residential, commercial, and industrial sources. Waste is typically transported to the landfill via open trucks and compactors and is subsequently dumped in designated areas. Given its critical role in the waste management system of Uyo, the Uyo Village Road landfill was deemed appropriate for conducting this study. The selection of this landfill as the study site was based on its operational characteristics, which are representative of other landfills in Nigeria. By selecting this location, the authors were able to obtain a representative sample of municipal solid waste that accurately reflects the overall composition of waste generated in the area and is essential for developing effective waste management strategies.

#### Waste characterization

Waste in this study was sampled using the quartering technique. This is a sampling method often used to sample heterogeneous materials such as municipal solid waste, often used when the sample is too large to be analyzed in its entirety. The entire sample is divided into four equal parts, and two opposing quarters are discarded while the remaining two quarters are combined and mixed. This process is then repeated until the desired sample size is achieved, which is usually a smaller, more manageable portion.

In this study, the waste samples were collected from “undisturbed waste,” immediately after it was unloaded at the landfill, this was carried out using a payloader. The sample size was critical in ensuring the accuracy and reliability of research findings. To obtain this, the sampling formula for continuous variable measurements (Eq. 1) was utilized^64^, which was applied by Gomez et al.^65^ and Miezah et al.^66^.

1 $$n = Z * Z\left[ {{{\left( {P * P} \right)} \mathord{\left/ {\vphantom {{\left( {P * P} \right)} {\left( {D * D} \right)}}} \right. \kern-0pt} {\left( {D * D} \right)}}} \right]$$

where *n* = the sample size, *Z* = value for a selected alpha level of each tail = 1.96; *P* = estimated population standard deviation based on a pre-study, and *D* = acceptable margin of error (0.05). From the calculation, the total waste analyzed was 9308.7 kg. The waste sample was manually divided by utilizing the coning and quartering method^67–69^. Here, the entire sample was mixed using a payloader and spread into a cone. The cone was then divided into four parts using a metal square pipe and spade. Two quarters, diagonally placed, were extracted and the remaining two quarters were mixed and quartered again. This procedure was repeated six times until the desired and manageable sample size of 120–150 kg was acquired. The characterization effort for this study was repeated over a period of seven days consecutively. As the desired sample size was obtained, the waste was moved from the main landfill to a nearby location for sorting and characterization.

#### Waste classification

For ease of recognition, the wastes in the landfill were classified by grouping similar wastes into the following groups:

i. Organic
ii. Plastics
iii. Paper
iv. Textiles
v. Ferrous and non-ferrous metals
vi. Glass
vii. Wood

#### Population determination and forecasting

Data published by the Nigerian National Bureau of Statistics^53^ on Nigeria’s population by region with forecast values up to 2033 was utilized in this study. These values served as basis for further prediction, and they are close to the values presented by PopulationStat^54^.

#### Determination of overall waste collection

Data on the average types and numbers of trucks that deliver waste to the site together with the average number of trips for each truck daily were recorded. To determine the total waste collected at the site, the weighbridge method was employed following global standards found in literature^55–57^. However, in absence of a weighbridge, the following equation (*Eq. *2) was employed to determine the overall quantity of waste collected.

2 $${{\text{MSW}}}_{{\text{col}}}= {\sum }_{j=1}^{365}{\sum }_{i=1}^{n}\left({C}_{Ti} x {V}_{Ri} x {\rho }_{i} x {t}_{nij}\right)$$

where:

$${{\text{MSW}}}_{{\text{col}}}=\mathrm{Quantity\, of \;MSW \;collected }\,({\text{tons}})$$

$${C}_{Ti}=\mathrm{Truck \;volume\; capacity} \,({m}^{3})$$

$${V}_{Ri}=\mathrm{Loading \;ratio\; per\; truck}$$

$${\rho }_{i}=\mathrm{Density \;of\; loaded \;MSW }\;(\frac{{\text{tons}}}{{m}^{3}})$$

$${t}_{nij}= \mathrm{ Number \;of\; trips\; per\; day}$$

Furthermore, the amount of waste generated per day was calculated based on a rule of thumb^58^, where approximately 74% of waste generated in developing countries is efficiently collected for disposal. Hence, a modification to Eq. 2 as presented by Ibikunle^59^ resulted in Eq. 3.

3 $${{\text{MSW}}}_{{\text{col}}}= {\sum }_{j=1}^{365}{\sum }_{i=1}^{n}\left({C}_{Ti} x {V}_{Ri} x {\rho }_{i} x {t}_{nij}\right) x \frac{100}{74}$$

MSW generation rate was estimated using Eq. 4 as presented by Atta et al.^60^

4 $${{\text{MSW}}}_{{\text{rg}}}=1000 x \frac{{{\text{MSW}}}_{{\text{tgen}}}}{{P}_{i} x 365}$$

where;

$${\text{MSW}}_{{{\text{rg}}}} = {\text{MSW generation rate}} \left( {\frac{{{\text{kg}}}}{{\frac{{{\text{person}}}}{{{\text{day}}}}}}} \right)$$

$$P_{i} = {\text{Population in the year}} i$$

$${\text{MSW}}_{{{\text{tgen}}}} {\text{ = Total MSW generated in year}} i$$

### Survey data collection

Google Forms was used to create online questionnaires that were accessible via a unique URL. Survey workers used either their mobile phones displaying the Google form, or paper survey forms with identical questions, to obtain survey responses from four different groups of Uyo residents: (a) people living in residential households more than a kilometer from the landfill, (b) residential households located within one kilometer of the municipal landfill, (c) market sellers at several markets with temporary stalls, and (d) employees at permanent businesses in buildings around town. There was a specific survey for each group, with some questions being identical. Grouping was done to assess whether different profiles of waste generation, and specific better options for waste management, exist in the context of this location. Data entered digitally by the surveyors into Google Forms was automatically recorded. Data recorded on the paper forms was entered manually into Google Forms according to each survey group, and automatically added to the other data from each respective group. As each questionnaire (digital or paper) was filled out face-to-face with the surveyor, there were no unanswered surveys. The time frame for each group was roughly one week in February 2023.

## Results and discussions

### Landfill operation and quantification of waste

**Figure 4:**
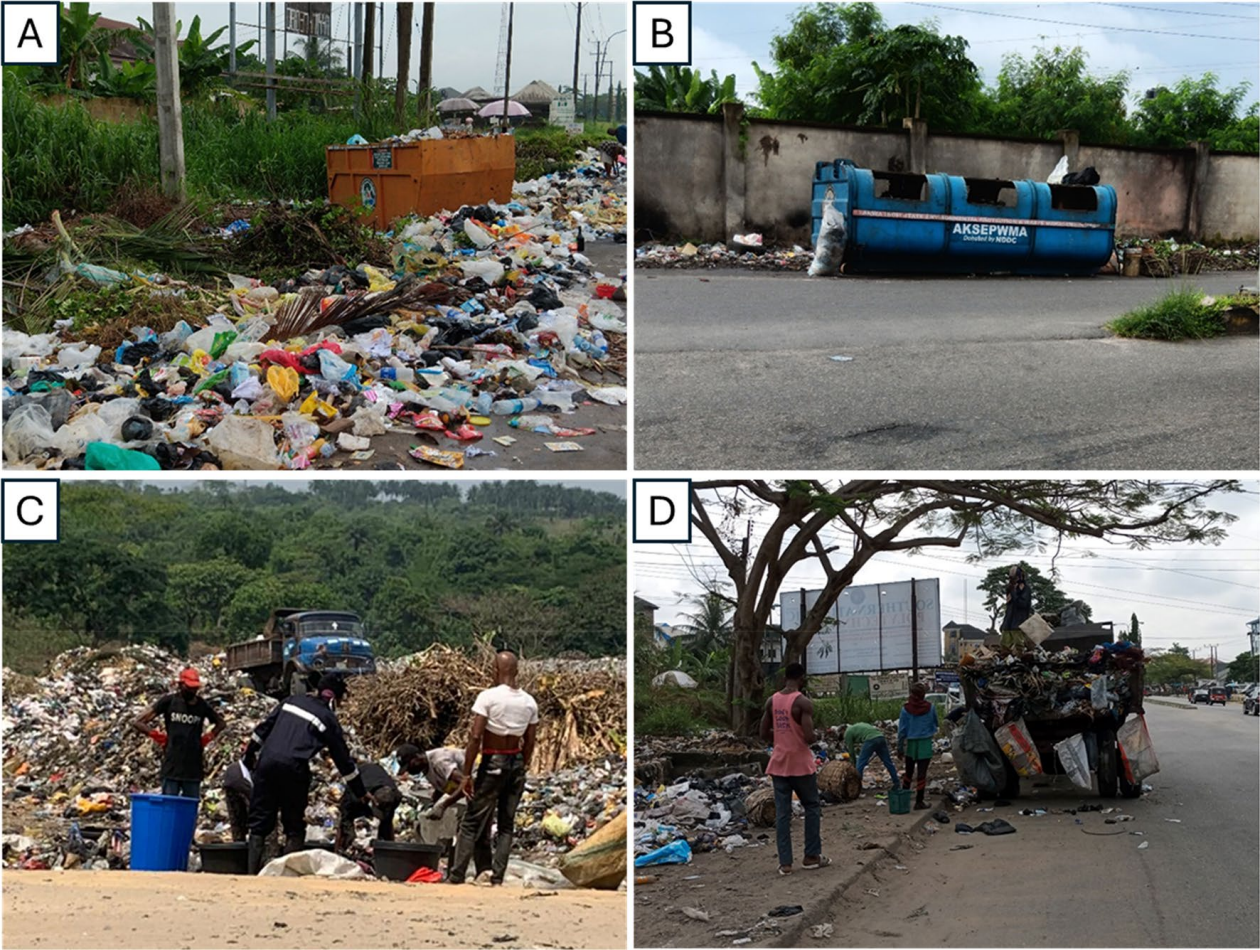
(**A**–**D**) Waste collection at various collection sites in Uyo.

The landfill is in operation from 6 AM to 6 PM daily. On a typical business day, the disposal facility closes to waste delivery trucks at 5 PM and the next hour is used for site-tidying activities. Typically, waste collection in the city begins early in the morning, typically at 7 AM. These generated wastes are dumped in publicly provided receptacles as presented in Figs. 4A–C. For collection, various types of vehicles such as compactors, tipper trucks, and utility vehicles are used (see Fig. 4D), which represent the origin of the waste. Specifically, compactors collect waste from the roadside, which is mainly around residential areas, whereas tipper trucks collect waste from the market area, while utility vehicles, also referred to as “house-to-house” collect waste from individual homes. The latter takes place only in high-income residential areas within Uyo, where residents are subscribed to a waste collection service either at a bi-weekly or monthly rate. At about 8:30–9:00 AM, the trucks start arriving at the landfill. Through interaction with the workers in the landfill, an estimated average of 30–50 trucks are emptied at the landfill daily. Similarly, each truck is expected to make an average of 3–5 trips daily, and this results from the high amount of daily waste generated in the city. At regular intervals within the day, already deposited solid waste is compacted using a bulldozer and a compactor. As is a common phenomenon in many developing countries, informal waste picking is carried out by people who scavenge through the waste stream in search of potentially valuable recyclable materials, such as scrap metal and plastic bottles, for the purposes of subsequent resale. The activities of these informal waste pickers have been critical in powering the recycling industry. At the landfill in Uyo Village Road, about 40 informal waste pickers rely on the collection and recovery of recyclable waste materials to support their livelihoods.

The total MSW collected and generated were calculated using Eqs. (2) and (3). Similarly, it was observed that each truck delivery is usually almost filled, but only to the brim of the lower end of the truck, and with uncompacted waste. Hence, it was assumed that trucks normally operate at 50% theoretical loading capacity. The result is presented in Table 2 below. The annual quantity of MSW generated in UCCDA, obtained by dividing by 0.74 based on the hypothesis that only 74% of waste is actually collected, was determined to be 690,541 tons. This figure represents approximately half of the MSW generated from more populous (approximately double) Nigerian states like Lagos and Kano considering dry seasons only. Hence, this presents an validation, and confirmation, of the effect of population on the quantity of MSW generated in a metropolis. Furthermore, the average MSW generation rate per capita was computed using Eq. 3; the value obtained was approximately 1.34 kg/person/day, which is in contrast to an approximate value of 0.66 kg/capita/day for comparable cities presented in literature a decade and a half ago. A reasonable explanation for this could be, among other factors, the increasing population size or the fact that residents of urban areas, as opposed to rural areas, tend to generate more recyclable waste that would end up in a landfill, rather than biodegradable waste that can be disposed of in nature^9,11,61^.

**Table 2:** Calculated quantity of MSW generated; the yearly amount of MSW collected is extrapolated from the number of tracks per day, their (used) capacity and the number of trips per day.

Truck type	Number of trucks per day	Capacity (t)	Volume capacity (m^3^)	Density of MSW (t/m^3^)	Loading ratio	Number of trips/day	Yearly MSW collected (t)	Yearly MSW generated (t)
Tipper Truck	20	20	16	1.25	0.5	4	292,000	394,595
Compactor	10	30	23	1.30	0.5	4	219,000	295,946
**Total**							**511,000**	**690,541**

**Figure 5:**
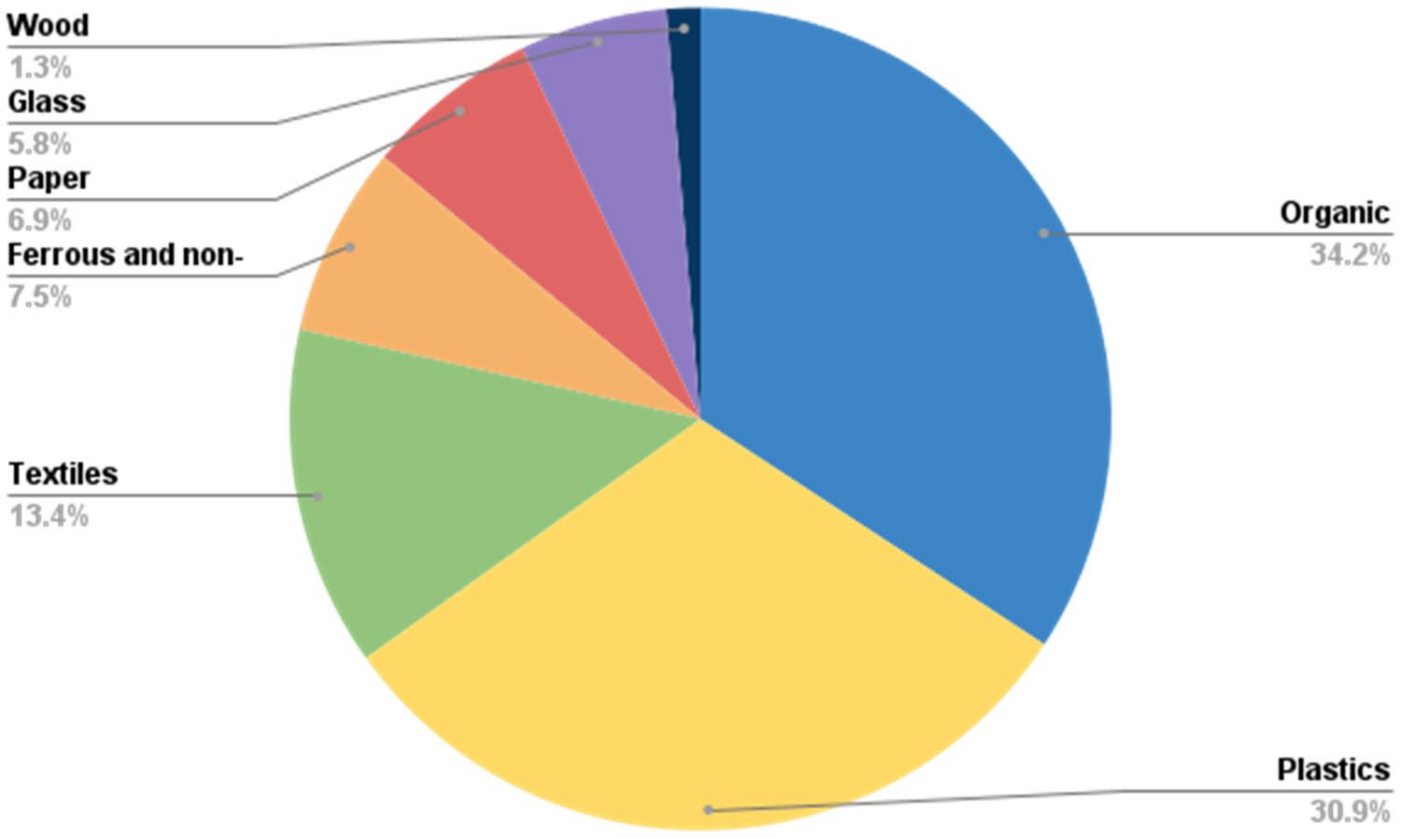
Waste composition in Uyo landfill serving UCCDA.

The MSW composition result of the landfill site obtained via the quaternary method described in the methodology is summarized in Fig. 5.

It is shown that the largest waste component of the landfill was mixed organic waste. This can partly be attributed to the agricultural and cultural pattern of the region, where agriculture is the dominant activity even among working-class households. On the other hand, the combined plastics and paper fractions made up approximately 38%. Partly, this can be attributed to the following: (1) increased packaging material consumption significantly influenced by increasing sales and trading activity dominant in the region, and (2) increasing population controlled by significant rural-to-urban migration rates experienced in recent times. Furthermore, from Fig. 5, the total recyclable solid wastes (plastics + metal + paper wastes) fraction exceeds 45% of the total waste. This presents an opportunity for integrating thermochemical waste conversion methods. Existing established methods provide an avenue to increasing the energy generation capacity of the region, noting that there is still insufficient consistent power supply for the entire region.

### Estimated waste generation forecast

By considering the waste generation rate for 2023, Fig. 6 presents the waste generation trend over the next ten years. The calculated yearly amount of waste generated in all of UCCDA was extrapolated based on the assumption of an annual population growth rate of 3.4%.

**Figure 6:**
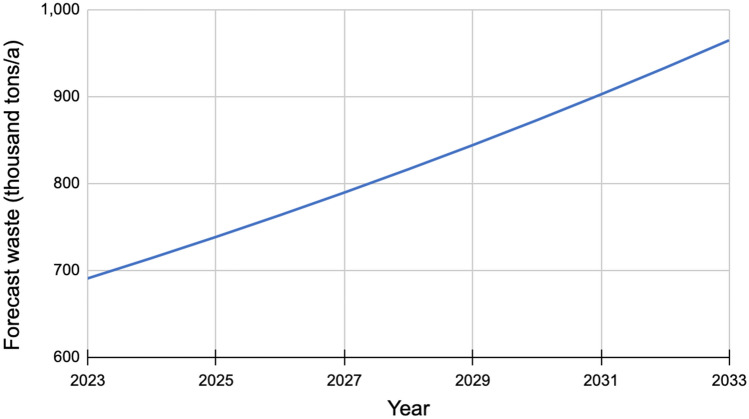
Yearly waste Generation Forecast in Uyo Capital City Development Area.

The forecast indicates that the amount of generated waste in ten years is bound to increase by approximately 40%, from 690,541 to 964,705 tons per year. Hence, adequate measures need to be put in place to ensure that these wastes are efficiently handled.

### Waste disposal survey

Four groups of people in the city of Uyo were questioned about their waste-disposing habits, composition of their waste, and the issues they are encountering among other things. The respondents were from residential households in average residential areas, residential households next to the landfill, market sellers at several markets with temporary stalls, and lastly employees at permanent businesses in buildings around town. The number of respondents in each group were 3632, 1407, 2019 and 2261, respectively, resulting in over 9000 completed surveys.

**Figure 7:**
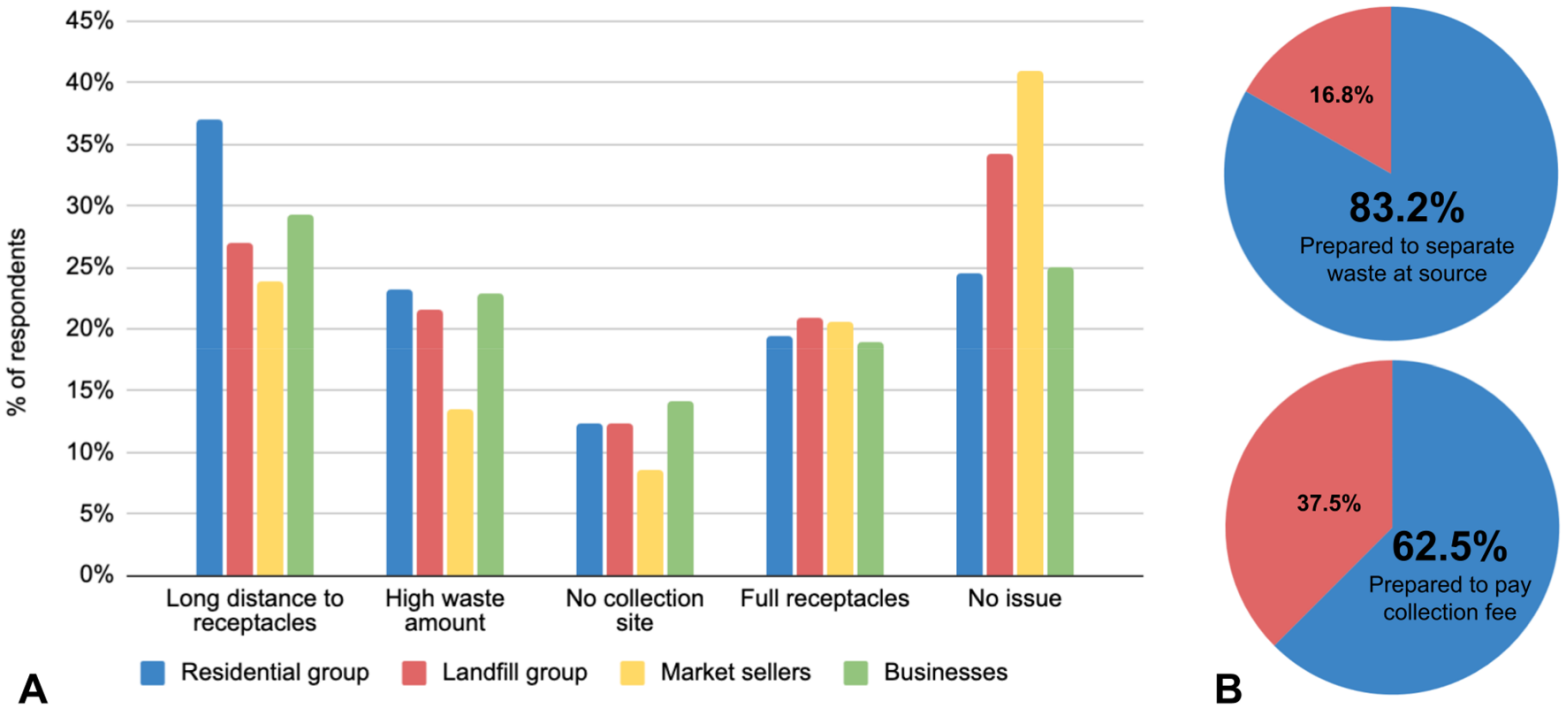
Survey responses from four different waste-producing groups in the city of Uyo; (**A**) main issues with waste disposal for the four different groups, in which more than one response was possible; (**B**) willingness of respondents to separate their waste at their homes or business sites, and to pay a fee for a government waste collection service.

Grouping the people polled allows for a more nuanced assessment of current issues and potential measures of addressing them. It was found that issues were shared between groups, but at different levels. Figure 7 shows selected results of this survey; firstly, from a multiple-choice question (“*What is the main problem you have disposing of your waste?*”), which posits that the main issues that respondents have with waste disposal in their respective situations (Fig. 7A) are presented. More than two thirds of respondents (68.8%) currently have one or more significant issues with waste disposal which are grouped as attitudinal (or willingness); namely, their waste collection point is too far away (‘distance’), they have too much waste to completely dispose of in an orderly fashion (‘waste amount’), they do not know where the closest collection site is located (‘no collection site’), or the site is always too full (‘full receptacles’). The foregoing agrees with a similar observation by Afroz et al^45^ where willingness to separate waste was traced to similar factors to those observed with our respondents. The distance from the disposal point is the biggest issue for average residents and businesses, with 37.0% and 29.3% citing this as their main issue; these same groups are most affected by the fact that there is too much waste for them to handle (23.2% and 22.8%, respectively). Roughly one fifth of all respondents encounter the problem of overflowing waste collection sites. The problem that seems to be the least prevalent is not knowing appropriate collection points to dispose of waste or the absence of a collection site, as less than 15% of respondents in each group named this factor. Still, this is a relevant factor that needs to be addressed. Market sellers reportedly had the least issues (41% responded not to have any problem), which ties in well with the fact that they dispose of their waste at the end of each day, and the collection site is always at the same market, if not very close to their stall. Residents at the landfill scored the second highest for this question, since they live very close to the waste disposal site.

Obtaining answers from discrete groups has the potential to provide better insights into how well certain waste management strategies will work to address the issues presented. It was found, however, that large parts of all groups were ready to cooperate with such measures. Two subsequent questions in the survey (*“If the government gave you two different bins (one for food waste, and one for everything else), to sort your waste into, would you sort it?”* and *“Would you pay a small fee if someone came to your house to collect your waste?”*) assessed the willingness of respondents from all groups to separate their waste at the source, and the willingness to pay a small fee for a waste collection service (Fig. 7B). The majority in each scenario were willing to cooperate (83.2% and 62.5%, respectively). This was in agreement with the result of a similar question posed by Patrick et al.^62^ using the same study area. However, there is the caveat that the cost of collection may not be affordable at present, and in the future, due to the rising cost of living (which is a factor affecting waste generation) as observed in literature^46–51^.

The issues the respondents have with waste disposal, and their readiness to support potential future efforts to curb these issues, suggest that better waste management practices through collecting waste closer to its source, then sorting and valorizing it, would be successful, and present a meaningful improvement in the livelihood of the people.

#### Statistical significance and analysis of survey responses

To observe if there were variations in challenges faced by respondents on existing waste management, an ANOVA test was utilized. Here, we determined if there were variations between responses obtained from the various survey groups (residential, landfill, market-sellers, businesses). Table 3 summarizes the percentage of responses obtained for each underlying issue.

**Table 3:** Summary of the percentage of responses obtained and grouped under waste management issues.

Response	Issues	Residential group	Landfill group	Market sellers group	Business place group
I have to carry it far	Long distance to receptacles	37.0%	27.0%	23.9%	29.3%
I have too much waste	High waste amount	23.2%	21.5%	13.5%	22.8%
I don't know where to take it (no collection bin)	No collection site	12.4%	12.4%	8.5%	14.2%
The central collection spot is always too full	Full receptacles	19.5%	20.9%	20.5%	19.0%
I don't have a problem	No issue	24.6%	34.3%	41.0%	25.0%

The analysis was premised on the following:

i. Both variables (dependent and independent) were independent of one another, hence, not skewed.
ii. There is homogeneous variation of the means for each set of data for all groups.
iii. The data were made up of independent observations.

The Null hypothesis (H_OS_) formed is:

**H**_**OS**_: *There is no variation among the respondent groups with respect to the waste management issues.*

The alternative hypothesis (H_OT_) is thus:

**H**_**OT**_: *There is a variation among the respondent groups with respect to the waste management issues.*

Additionally, the analyses were performed with the significance value, α, set at 5% (0.05), which signifies that the permissible upper limit of the risk associated with rejecting a true null hypothesis. The ANOVA revealed that there was no statistically significant difference in the responses. This is indicated by the small F-value and high *P*-value > 0.05) in Table 4, which summarizes the ANOVA statistical values. Hence, we will fail to reject the Null hypothesis, proving that there was no statistical significance variation. This means that all the survey groups faced similar challenges with the existing waste management.

**Table 4:** ANOVA results.

Source of Variation	SS	df	MS	F	*P*-value	F crit
Between Groups	0.001228	3	0.000409	0.048944	0.985138	3.238872
Within Groups	0.133786	16	0.008362			
Total	0.135014	19				

### Proposed waste management options

**Figure 8:**
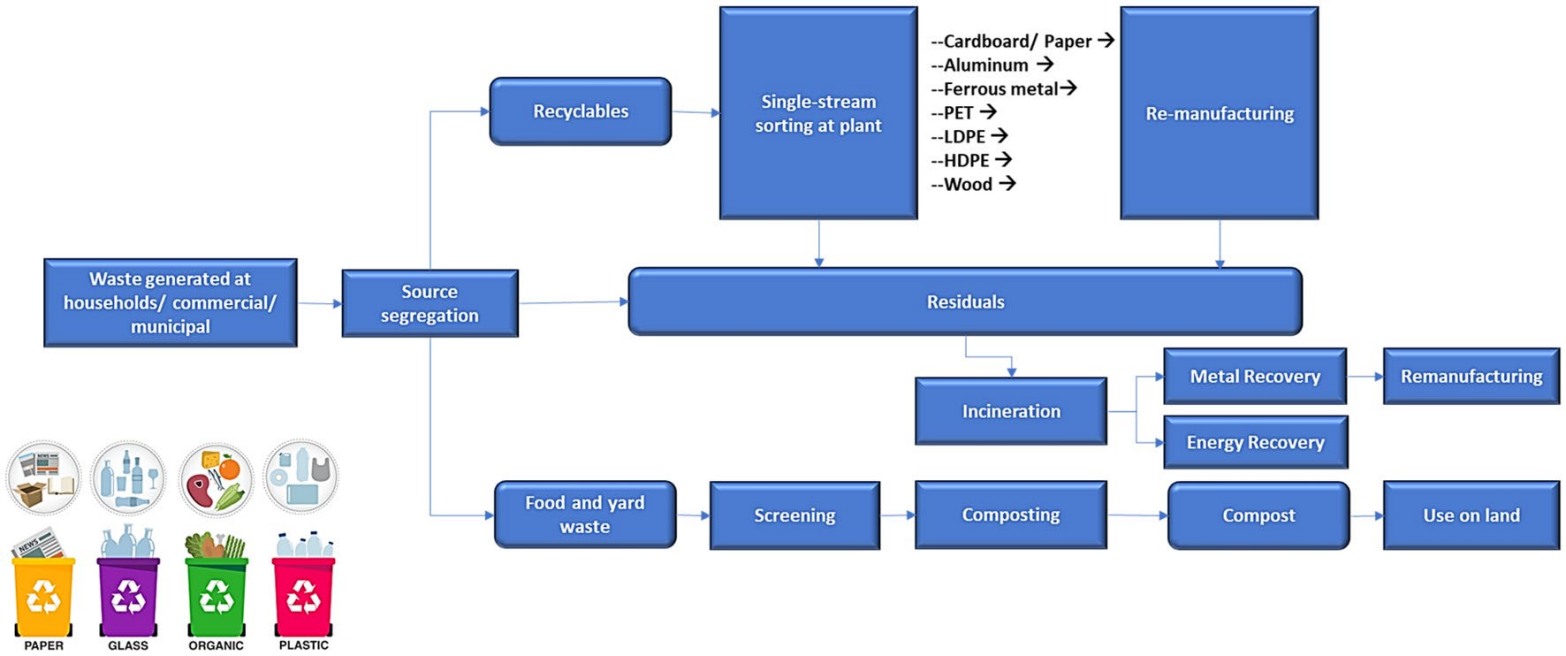
Schematic of the proposed improved waste management in Uyo.

As the current waste management system practiced in the region involves manual handling, inefficient collection and sorting, limited recycling, and landfilling as final disposal method, present-day developments in waste management strategies hold better opportunities for valorization of the waste generated in the region. With the forecast waste quantities projected to increase by approximately 40% (see Fig. 6), there is a need to propose a more efficient and proper management strategy. Figure 8 summarizes a more valuable technique with potential opportunities for revenue generation.

#### Waste collection and sorting

One of the challenges typically faced by waste handling facilities is the problem of mixed or combined waste fractions. This becomes increasingly challenging when dealing with waste in bulk quantities such as in large landfills like the one operated by the region in view. Scaling down waste sorting and relegating the sorting process to the source is one way to ease the process. Hence, a subscription-based model should be adopted which strictly requires that households in the region sort their wastes into different collection bins. The sorted fractions can be according to their recyclability, that is, food waste, recyclable, non-recyclable and hard paper/carton. Such a model will be convenient and offer several advantages over the current general city-wide waste collection. Additionally, monetary fines by the collection service can be implemented to ensure compliance, which is an incentive for proper waste sorting.

#### Valorization

**Figure 9:**
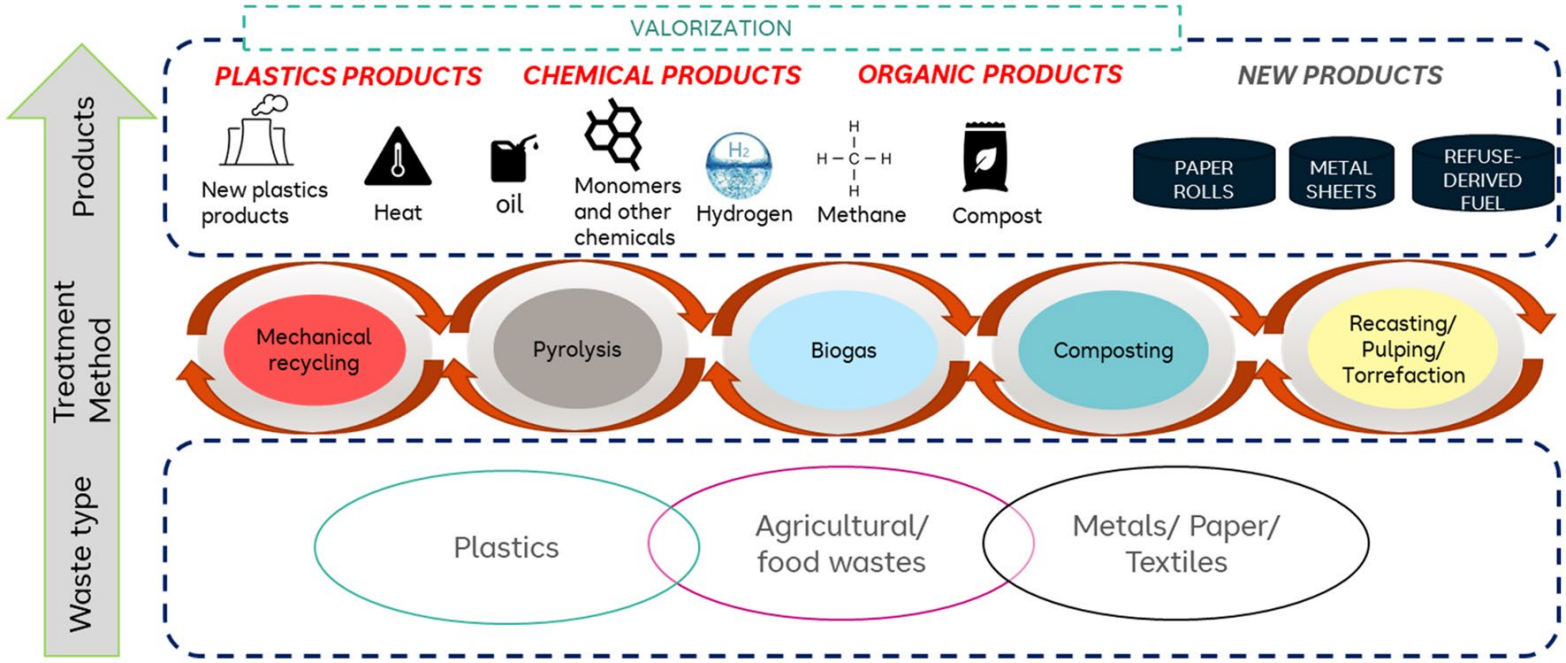
Simplified summary of the waste valorization methods.

Waste valorization has become integral, with a focus on attaining global sustainability in 2030. For the region in view, the valorization methods employed are reusing and partly downcycling. Here, homes practice the reuse of glass or tin packages to store food or other items, especially in the rural areas. Also, local waste pickers scavenge through the waste stream at the landfills, in search of potentially valuable recyclable materials, such as scrap metal and plastic bottles, for the purposes of subsequent resale to mechanical recyclers. The downstream use of these resold materials usually involves reuse for the same purpose or downcycling for lower grade materials. This mostly involves non-transformation of the chemical state of the materials. However, these efforts by waste pickers are insufficient to effectively reduce the quantity of available waste in the landfills. Hence, other valorization methods are desirable, which are summarized in Fig. 9.

#### Waste-to-energy recovery

This involves thermochemical conversion of the waste materials into other chemical products. It can be employed to generate more valuable products, especially those with high energy content which could help to address the power shortage experienced in the region. As reviewed earlier, thermochemical valorization processes include pyrolysis, gasification, and others. The significant amount of plastics fraction in the waste characterization results (*see *Fig. 5) presents enormous potential for setting up medium-scale thermochemical conversion plants. This could be based on the aforementioned processes, where the rejected fractions are utilized as feedstock to produce high-energy products like bio-oils, biofuels, syngas, and pyrolytic oil, thereby supplementing energy for the region. These technologies have been extensively reviewed in literature, and there exist several process technology licensors and plants in operation^13,63^.

#### Waste-agriculture integration

The organic waste fraction in the landfill is composed mainly of food wastes from the restaurants, markets and homes. These organic wastes undergo continuous decomposition, though at a slow rate, but the compost is not utilized in any form. Hence, one proposal would be to collaborate with the agricultural sector to develop proper composting dumps integrated with large scale commercial farming in the region. These commercial farms could generate income from sales or generate feedstock for small and medium enterprise-based manufacturing facilities.

## Conclusion and recommendations

This article was focused on characteristics and management of municipal solid waste in Uyo, Akwa Ibom state, Nigeria. The current waste management system in Uyo was assessed, a sampling design performed, an estimated waste generation forecast was calculated, and improved waste management options were identified based on the waste characterization and results from surveys. Hence, the following conclusions were drawn:

- Plastic, paper, glass and metal wastes made up over half (> 51%) of the waste collected in Uyo municipality, meaning there is a large potential of valorizing the recyclable fraction of the waste.
- The current waste management approach is inefficient in handling the quantity of waste generated in the municipality, most of which is disposed of in the landfill. This will be exacerbated in ten years, at which point potential waste generation is estimated to increase by 40%.
- Currently, most of the potential of the waste is lost in the landfill. However, an enormous energy and revenue generation potential exists if the strategies outlined in the previous section are properly harnessed.

It is imperative to gradually reduce and eliminate the landfilling system. This can be achieved through synergy between private actors and the municipality. In addition, incentivization strategies need to be developed to encourage the citizens to participate in an integrated waste management scheme.

### Ethical approval

Ethical approval was not required for this study.

### Consent to participate

Survey participants were informed on the purpose of the survey as follows, ‘This is an anonymous survey to help inform our state government on the needs of the citizens of Uyo with regards to their waste. The survey is being conducted privately. It contains less than ten questions about your experience managing your waste and you are free to participate as you choose.’ Verbal consent was then given by survey participants.

### Supplementary Information

Supplementary Information.

## Data Availability

The original data used in this work is available upon request. This can be requested from: Corresponding author: Uduak Bassey. Email: uduak.bassey@srh.de.
