# Supplementary material for: Characteristics and management of municipal solid waste in Uyo, Akwa Ibom state, Nigeria
Source: Sci Rep. 2024 May 14;14:10971. doi: 10.1038/s41598-024-61108-0 (PMC11094142; doi:10.1038/s41598-024-61108-0)
Supplement: Supplementary file 1 — Supplementary Information. [file 41598_2024_61108_MOESM1_ESM.docx]

**Appendix**

Supplementary Table 1: Population from Nigerian national census in 2006, and projected population in 2023, as well as percentage estimates of LGAs surrounding Uyo LGA which contribute to the waste at the landfill studied (Source: Nigerian National Bureau of Statistics, 2006; Ministry of Economic Development Uyo, Akwa Ibom State annual report, 2007).

| **LGA** | **Area (km² )** | **Population 2006** | **Projected population 2023** | **UCCDA Assumption** | **Population (UCCDA)** |
| --- | --- | --- | --- | --- | --- |
| Uyo | 362.00 | 305,961 | 540,151 | 100% | 540,151 |
| Nsit Ibom | 144.60 | 108,095 | 190,834 | 30% | 57,250 |
| Itu | 195.30 | 127,856 | 225,720 | 50% | 112,860 |
| Ibesikpo | 175.50 | 137,127 | 242,087 | 40% | 96,835 |
| Abak | 190.00 | 139,069 | 245,516 | 50% | 122,758 |
| Etinan | 157.20 | 168,924 | 298,223 | 40% | 119,289 |
| Uruan | 449.00 | 117,169 | 206,853 | 50% | 103,427 |
| Ikono | 197.40 | 131,673 | 232,459 | 40% | 92,983 |
| Ibiono Ibom | 336.00 | 188,605 | 332,968 | 50% | 166,484 |
|  |  |  |  |  |  |
| **Total population** |  |  | 2,514,810 |  | 1,412,037 |

Supplementary Table 2: List of questions used in the survey. All were single- or multiple choice questions; some response options differed between groups; a checkmark denotes that the questions

| **Question** | **Group A Residential group** | **Group B Landfill group** | **Group C Market sellers** | **Group D Businesses** |
| --- | --- | --- | --- | --- |
| How do you dispose of your waste at the moment? | ✔ | ✔ | ✔ | ✔ |
| How happy are you with how you dispose of your waste at the moment? | ✔ | ✔ | ✔ | ✔ |
| What is the main problem you have disposing of your waste? | ✔ | ✔ | ✔ | ✔ |
| What type of waste do you mainly produce? | ✔ | ✔ | ✔ | ✔ |
| If the government gave you two different bins (one for food waste, and one for everything else), to sort your waste into, would you sort it? | ✔ | ✔ | ✔ | ✔ |
| How much waste do you produce per week? | ✔ | ✔ | ✔ | ✔ |
| How often do you take out your waste? | ✔ | ✔ | ✔ | ✔ |
| Would you pay a small fee if someone came to your house to collect your waste? | ✔ | ✔ | ✔ | ✔ |
| How much money would you pay per month for this service? | ✔ | ✔ | ✔ | ✔ |
| Are you dealing with health problems because the landfill is near your house? |  | ✔ |  |  |
| Which of these symptoms do you have often? |  | ✔ |  |  |
| What is your experience with flies, insects, cockroaches and rats? |  | ✔ |  |  |
| What would help you deal with your waste better? |  |  | ✔ |  |
